# Titanium Elastic Nailing Versus Hip Spica Cast in the Treatment of Femoral Shaft Fractures in Children

**DOI:** 10.7759/cureus.83868

**Published:** 2025-05-10

**Authors:** Habiba Ijaz, Hussain Ali, Muhammad Mannan, Muhammad Awais Iqbal, Muhammad A Hamid

**Affiliations:** 1 Trauma and Orthopaedics, Kettering General Hospital NHS Trust, Kettering, GBR; 2 Orthopaedic Surgery, University Hospitals Birmingham, Birmingham, GBR; 3 Orthopaedics and Trauma, The Dudley Group NHS Foundation Trust, West Midlands, GBR; 4 Trauma and Orthopaedics, Ghurki Trust Teaching Hospital, Lahore, PAK

**Keywords:** alignment, callus, femoral shaft fractures, hip spica cast, skin traction, titanium elastic nailing, weight bearing

## Abstract

Objective

The objective of this study is to compare titanium elastic nailing (TEN) to hip spica casting (HSC) in the treatment of paediatric femoral shaft fractures, focusing on hospital stay duration and time to initiation of weight bearing.

Study design

This was a quasi-experimental study conducted at the Department of Orthopaedic Surgery, Benazir Bhutto Hospital, Rawalpindi, over a six-month period.

Duration and place of study

The data for this study were collected at the Department of Orthopaedic Surgery, Benazir Bhutto Hospital, a major tertiary care and trauma referral centre, over a six-month period from 8 August 2018 to 8 February 2019.

Patients and methods

A total of 60 children aged 6-12 years with femoral shaft fractures were enrolled using consecutive sampling and divided into two groups based on the treatment provided by the consultant orthopaedic surgeon. Group A (n=30) received titanium elastic nailing, while Group B (n=30) underwent early hip spica casting after initial skin traction. Follow-ups were conducted at two, four, six, eight and 12 weeks to evaluate hospital stay, time to weight bearing and bone healing. Clinical assessment included the range of motion and pain evaluation to guide weight bearing. Data were analysed using SPSS version 22 (IBM Corp., Armonk, NY); means and standard deviations were calculated for quantitative variables, and independent t-tests were used for group comparisons. Stratification was done for age, gender and fracture side, with a p-value of <0.05 considered statistically significant.

Results

In Group A (TEN group), the mean duration of hospital stay was 3.93±0.78 days, compared to 3.03±1.03 days in Group B (HSC group) (p<0.05). The time to start weight bearing was 30.57±6.81 days in Group A and 59.5±14.03 days in Group B (p<0.05). In the TEN group, surgery was typically performed within 48-72 hours of injury, depending on patient stabilisation and operating room availability. In the HSC group, hip spica casting was applied within 72 hours of injury following initial skin traction. Weight-bearing decisions in both groups were based on a combination of clinical evaluation, including the absence of pain on movement and the restoration of joint mobility, and the radiological evidence of bone healing, defined as callus formation on at least three out of four cortices on anteroposterior (AP) and lateral radiographs.

Conclusion

This study observed that titanium elastic nailing was associated with the earlier initiation of weight bearing compared to hip spica casting in children aged 6-12 years with femoral shaft fractures. As hip spica casting is more commonly recommended for younger children under five years of age, our findings suggest that titanium elastic nailing may be a more suitable option in older paediatric patients, particularly in settings where surgical infrastructure is available. While TEN demonstrated potential advantages in terms of earlier functional recovery, it is important to interpret these findings within the context of the study's focus on time to weight bearing and hospital stay.

## Introduction

Children are more likely to suffer a shaft of femur fracture due to their active lifestyle, anatomical configuration and biomechanical differences from adults [1]. These injuries require hospitalisation. The healing of such injuries is very rapid due to the rich blood supply of the femoral shaft. A trend towards operative stabilisation is seen for children with femoral shaft fractures, depending on their age and the type of injury [2]. Preschool-aged children still benefit from nonsurgical treatment not just because it is cost-effective and commonly preferred but also due to their high remodelling potential, smaller size and better tolerance to immobilisation methods such as traction and spica casting. Additionally, non-operative management avoids surgical and anaesthesia-related risks in this younger age group.

In the case of a school-age child with an isolated femoral shaft fracture, surgical treatment is generally preferred due to more rapid mobilisation and the earlier resumption of daily activities. Titanium elastic nailing (TEN) has demonstrated significant benefits in children aged 6-16 years. In addition to being easy to use, it is less invasive and provides excellent functional outcomes [3,4]. Hip spica casting (HSC), however, remains a widely used treatment modality in paediatric orthopaedics, despite the growing preference for operative interventions such as TEN [5].

A study by Shemshaki et al. demonstrated that TEN results in significantly shorter hospital stays (6.9±2.9 days) and earlier mobilisation with aids (17.6±10.2 days) compared to hip spica casting (20.5±5.8 days) (p<0.001) [6]. While international studies have shown the benefits of TEN, there is comparatively limited literature on its use in the Pakistani paediatric population. However, some local studies have reported similar findings, indicating shorter recovery and earlier weight bearing with TEN compared to spica casting [7]. These findings highlight the importance of context-specific research, as ethnic, geographical and healthcare system differences may influence outcomes. Our study aimed to contribute to this limited local evidence by comparing titanium elastic nailing to hip spica casting in terms of hospital stay and time to weight bearing in children with femoral shaft fractures.

## Materials and methods

A quasi-experimental prospective study targeting paediatric patients with femoral shaft fractures was conducted at the Department of Orthopaedic Surgery, Benazir Bhutto Hospital, Rawalpindi, Pakistan. The study specifically included children aged 6-12 years and was conducted following ethical approval from the Research Evaluation Unit of the College of Physicians and Surgeons Pakistan (CPSP) (approval number: CPSP/REU/OSG-2017-126-1818). The data collection period spanned from 8 August 2018 to 8 February 2019. The study aimed to compare two different treatment methods, titanium elastic nailing and hip spica casting, in terms of hospital stay and time to initiation of weight bearing.

The sample size was calculated using data from a previously published study, which reported a mean time to early weight bearing of 17.6 days in Group A and 65.6 days in Group B, with a standard deviation of 10.45. Based on these values and using a 99% power and a 5% level of significance, the required sample size was determined to be 30 patients per group. A total of 60 children, aged 6-12 years and of both genders, were enrolled using a non-probability consecutive sampling technique from the Orthopaedic Surgery Department of Benazir Bhutto Hospital, Rawalpindi. Children with open fractures, comminuted femoral shaft fractures, bleeding diathesis or pathological fractures were excluded, as these conditions required alternative management strategies.

Patients were divided into two groups based on the treatment approach decided by the consultant orthopaedic surgeon, taking into account the patient's age, fracture characteristics and overall clinical condition. Group A (titanium elastic nailing) included 30 children who underwent operative treatment with titanium elastic nails, while Group B (hip spica cast) consisted of 30 children who received early hip spica casting following initial traction. Informed written consent was obtained from the parents or guardians of all participants before their inclusion in the study.

For Group A, pre-anaesthesia workup was completed in all patients. The surgical procedure was performed under general anaesthesia by a consultant orthopaedic surgeon with over four years of experience, assisted by a trainee registrar. Titanium elastic nails were inserted retrograde through small incisions under image intensifier guidance, extending proximally to the physeal zone. Fracture reduction was confirmed by achieving proper alignment and length. Nail diameter was selected based on the width of the medullary canal at its narrowest part. Postoperative antibiotics were administered, and patients were discharged once stable. The hospital stay duration was recorded, and patients were reviewed at two weeks for wound inspection, stitch removal and postoperative X-rays. Weight bearing was typically initiated at four weeks post-surgery.

For Group B, early hip spica casts were applied following initial skin traction, typically within 72 hours of injury. The cast application was performed under monitored conditions in the operating theatre. Sedation was administered using oral midazolam (0.5 mg/kg) and syrup paracetamol (15 mg/kg) approximately 30 minutes before the procedure, under the supervision of an anaesthetist. A one-and-a-half spica cast was applied, extending from the chest to the distal tibia on the affected leg and to the distal femur on the contralateral side. Alignment was maintained using traction and fluoroscopic guidance. After the confirmation of satisfactory alignment, the patient was discharged with cast care instructions. A follow-up was conducted at two weeks to evaluate the cast condition and fracture alignment. The cast was maintained for six weeks in children under 10 years and for eight weeks in those aged 10 years or older. After cast removal, physiotherapy was initiated, and weight bearing was gradually encouraged.

Both groups were followed up at two, four, six, eight and 12 weeks post treatment. At each follow-up, assessments were performed in the outpatient clinic by a consultant orthopaedic surgeon and a trainee registrar in the presence of the child's parents. Clinical evaluation included the range of motion, pain on movement and stability at the fracture site. The timing of weight-bearing initiation was determined based on the absence of pain, the restoration of joint mobility and the treating surgeon's clinical judgement.

Data were analysed using SPSS version 22 (IBM Corp., Armonk, NY). Quantitative variables such as age, hospital stay duration and time to weight bearing were expressed as means and standard deviations. Qualitative variables such as gender and fracture side were presented as frequencies and percentages. An independent samples t-test was applied to compare mean values between the two groups. Baseline characteristics were reviewed to assess comparability, and stratification was done for variables such as age, gender, side of fracture and mode of injury. A p-value of <0.05 was considered statistically significant.

## Results

The data were collected using a specially designed pro forma. In the total study population, 66.7% (n=40) of patients were men with a mean age of 8.07±2.08 years, while 33.3% (n=20) of patients were women with a mean age of 8.05±1.54 years. There were equal numbers of men (n=20) and women (n=10) in both groups, while in Group A (TEN group), the mean age was 8.43±1.92 years, and in Group B (HSC group), it was 7.70±1.84 years. A detailed demographic profile of both groups is shown in Table 1.

**Table 1:** Demographic profile of femoral shaft fracture in both groups TEN, titanium elastic nailing; SD, standard deviation

Group	Category	n (%)	Mean Age±SD (Years)
Group A (TEN group)	Male	20 (66.7%)	8.45±2.06
	Female	10 (33.3%)	8.40±1.71
	Total	30 (100%)	8.43±1.92
Group B (hip spica group)	Male	20 (66.7%)	7.70±2.08
	Female	10 (33.3%)	7.70±1.34
	Total	30 (100%)	7.70±1.84

**Figure 1:**
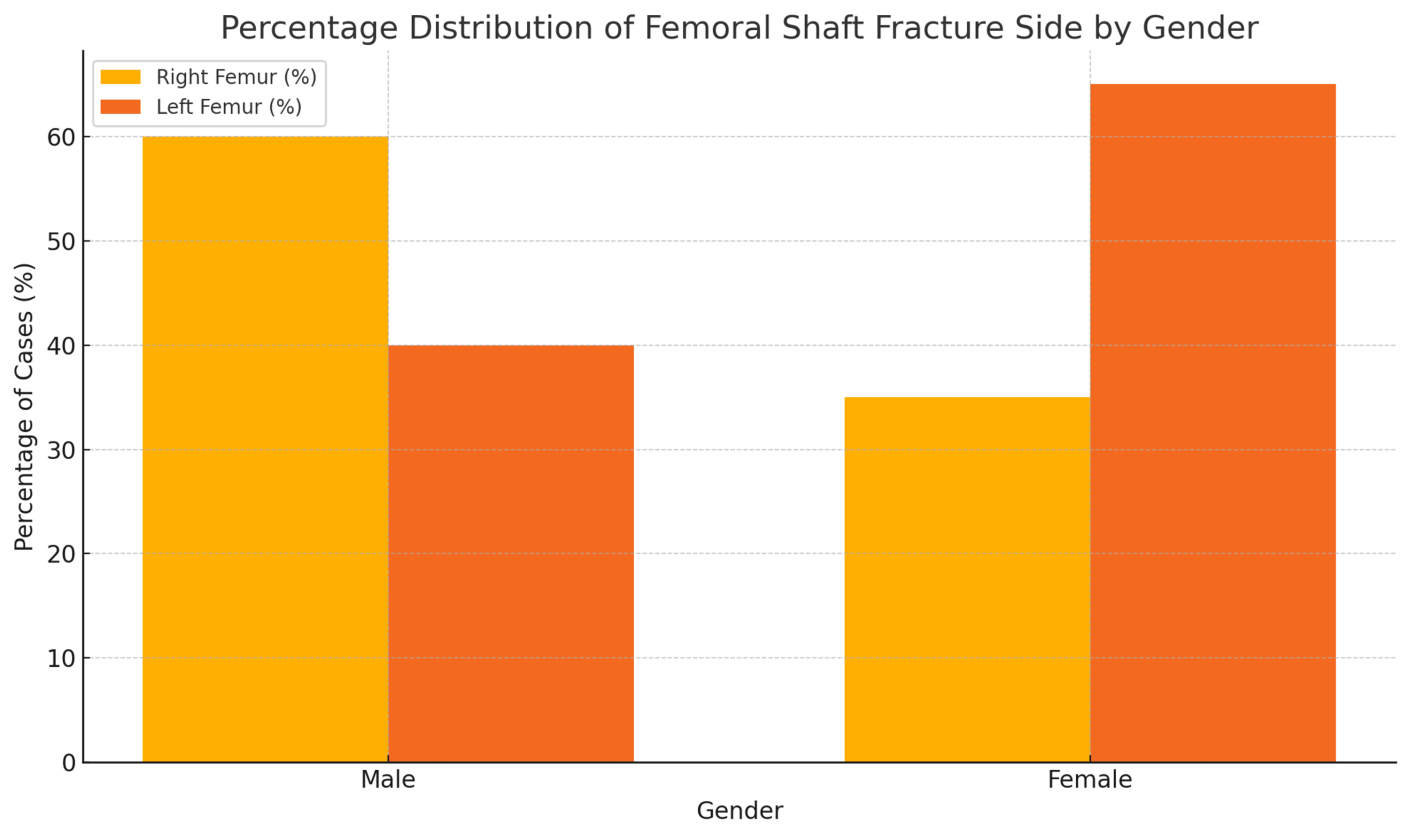
Percentage distribution of right and left femoral shaft fractures according to gender

Out of a total of 60 children with femoral shaft fractures, 51.7% (n=31) presented with right femoral shaft fractures, and 48.3% (n=29) presented with left femoral shaft fractures. A detailed demographic profile in accordance with the side of the fractured femoral shaft is described in Figure 1.

**Figure 2:**
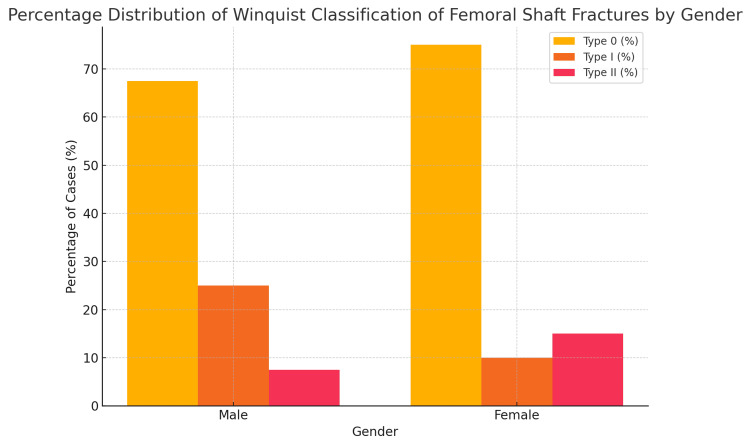
Percentage distribution of Winquist classification types of femoral shaft fractures according to gender

A total of 42 out of 60 patients (70%) presented with Winquist type 0 fracture, while Winquist type I and II fractures were present in 12 (20%) and six (10%) patients, respectively. This is presented in Figure 2.

**Figure 3:**
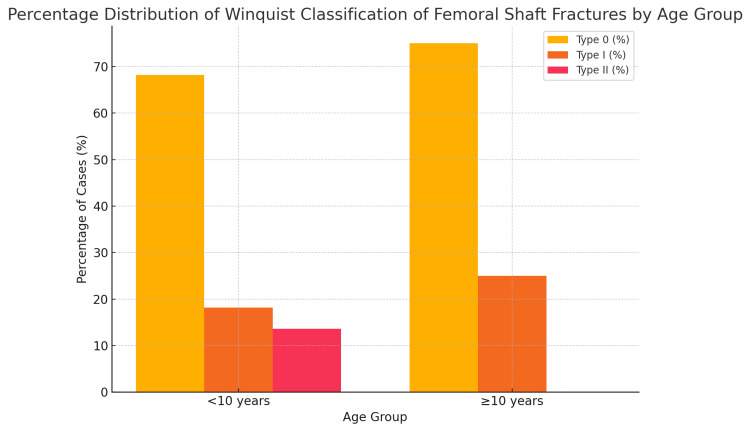
Percentage distribution of Winquist classification types of femoral shaft fractures according to age group

Among children aged less than 10 years, Winquist type 0 fractures accounted for the majority, followed by type I and type II fractures. In children aged 10 years and older, type 0 fractures also predominated, while no cases of type II fractures were observed in this group. These findings suggest that simpler fracture patterns (type 0) were the most common in both younger and older age groups, with more complex fracture patterns (type II) occurring exclusively in children under 10 years, which is presented in Figure 3.

This prospective study found a statistically significant difference in both hospital stay duration and time to initiation of weight bearing between the two treatment groups. When stratified by age, gender and fracture side (Table 2), titanium elastic nailing consistently resulted in significantly earlier initiation of weight bearing compared to hip spica casting across all subgroups. In children aged less than 10 years, the mean time to weight bearing was 28.95±6.6 days in the TEN group versus 55.29±12.1 days in the HSC group (p=0.032), while in those aged 10 years or older, it was 33.80±6.3 days and 76.33±6.5 days, respectively (p=0.028). Among male patients, weight bearing was initiated at 29.40±6.6 days in the TEN group compared to 60.50±14.9 days in the HSC group (p=0.041), and among female patients, weight bearing was initiated at 32.90±7.0 days versus 57.50±12.6 days, respectively (p=0.039). Similarly, for right-sided femoral fractures, weight bearing commenced at 29.40±6.5 days in the TEN group and 62.00±15.4 days in the HSC group (p=0.036), while for left-sided fractures, it began at 31.73±7.1 days compared to 56.64±12.3 days, respectively (p=0.034). The analysis of hospital stay stratified by Winquist fracture type (Table 3) showed that in patients with type 0 fractures, the TEN group had a significantly longer hospital stay (3.95±0.89 days) compared to the HSC group (2.82±0.91 days) (p=0.045), while no statistically significant differences in hospital stay were observed in type I or type II fractures (p>0.05). These findings confirm that titanium elastic nailing consistently promoted earlier weight bearing across all age groups, genders and fracture sides, while hospital stay was slightly longer in TEN-treated simple fractures (type 0) as detailed in Table 2 and Table 3.

**Table 2:** Mean time to weight bearing in both groups (age, gender and femoral shaft fracture side-based stratification) (n=60) Independent Student's t-test SD: standard deviation

Group	Treatment Group	Time to Weight Bearing (Mean±SD, Days)	P-value
<10 years	Titanium elastic nailing	28.95±6.6	0.032
	Hip spica cast	55.29±12.1	0.032
≥10 years	Titanium elastic nailing	33.80±6.3	0.028
	Hip spica cast	76.33±6.5	0.028
Male	Titanium elastic nailing	29.40±6.6	0.041
	Hip spica cast	60.50±14.9	0.041
Female	Titanium elastic nailing	32.90±.0	0.039
	Hip spica cast	57.50±12.6	0.039
Right	Titanium elastic nailing	29.40±6.5	0.036
	Hip spica cast	62.00±15.4	0.036
Left	Titanium elastic nailing	31.73±7.1	0.034
	Hip spica cast	56.64±12.3	0.034

**Table 3:** Mean duration of hospital stay in both groups (stratification on the basis of Winquist type) (n=60) Independent Student's t-test SD: standard deviation

Winquist Type	Treatment Group	Duration of Hospital Stay (Mean±SD, Days)	P-value (T-test)
Type 0	Titanium elastic nailing	3.95±0.89	0.045
	Hip spica cast	2.82±0.91	0.045
Type I	Titanium elastic nailing	4.00±0.58	0.078
	Hip spica cast	3.60±1.34	0.078
Type II	Titanium elastic nailing	3.67±0.58	0.092
	Hip spica cast	3.67±1.15	0.092

## Discussion

The malunion or maltreatment of a femoral fracture may result in complications such as shortening, deformity, delayed healing, nonunion and infections. Traditionally, spica casting or traction, followed by casting, has been the standard treatment for paediatric diaphyseal femur fractures, as recommended by the American Academy of Orthopaedic Surgeons (AAOS), particularly for children aged six months to five years [8]. However, a shift towards operative approaches has occurred in older children, with flexible intramedullary nails such as titanium elastic nails (TEN) gaining preference due to their minimally invasive nature, biomechanical stability and effectiveness in promoting rapid healing [9,10]. Unlike more invasive techniques such as external fixation or plate fixation, TEN preserves the femoral blood supply and facilitates early mobilisation, reducing complications associated with prolonged immobilisation, such as limb length discrepancies. Additionally, early discharge associated with TEN can reduce the economic burden on families due to decreased hospital stays.

Our study aims to compare two commonly used treatment modalities, titanium elastic nailing and early hip spica casting, in terms of hospital stay and time to weight bearing in children aged 6-12 years. The findings support the hypothesis that TEN offers functional advantages over hip spica casting. Specifically, patients in the TEN group began weight bearing significantly earlier and had a slightly longer hospital stay, largely attributable to the surgical nature of the intervention. These results directly address the research objective and demonstrate clinically relevant benefits of TEN in terms of early mobilisation and return to daily activities.

Our findings are consistent with both national and international literature. Verma et al. [11] and Saseendar et al. [12] also reported shorter time to weight bearing and lower complication rates in patients treated with TEN compared to hip spica casts [13,14]. Saseendar et al. found that fracture union occurred earlier in the surgical group (six weeks) than in the spica group (eight weeks), with a p-value of 0.001. Spica castings resulted in significant coronal plane angulation (p<0.001), prolonged immobilisation (p<0.001) and later full weight bearing (p<0.001). Compared to hip spica casting, titanium elastic nailing resulted in better Flynn scores and earlier functional recovery [12].

A study conducted by Mehdinasab et al. evaluated and compared the short-term results of hip spica casts and intramedullary pin fixations in children aged 6-12 years [15]. The average treatment duration from admission to independent walking was 75.3 days for the spica cast group and 61.2 days for the TEN group (p<0.05). Hossain et al. also found that intramedullary fixation by TEN was effective in properly selected patients aged 5-15 years in Bangladesh [16]. A retrospective comparative study by Nascimento et al. reported a mean hospital stay of nine days for the TEN group and 20 days for the HSC group (p<0.005) [17]. Patients in the TEN group began weight bearing after 3.5 weeks, compared to 9.6 weeks in the HSC group. Similarly, Khan et al. demonstrated that retrograde flexible intramedullary nails were more effective than immediate hip spica casts in treating paediatric diaphyseal fractures in Pakistani patients, with earlier functional recovery and earlier weight bearing (p<0.005) [18].

The consistent advantage of TEN across various studies, including ours, highlights its effectiveness across diverse patient profiles such as age, gender, fracture side and Winquist classification. Nonetheless, we acknowledge that the non-randomised nature of our study may introduce selection bias, as treatment allocation was based on the attending surgeon's clinical judgement. Factors such as fracture stability, patient nutritional status and socioeconomic considerations may have influenced the decision to opt for TEN or spica casting, potentially impacting the comparability between groups.

No major complications such as infection, reoperation, neurovascular injury or skin-related issues associated with spica casting were observed in either group during the follow-up period of this study. Some patients did experience minor complications, including malunion and leg length discrepancy; however, these did not result in any significant functional limitations and did not necessitate additional intervention. While these findings are encouraging, the formal documentation and analysis of such complications were not part of the study protocol. Future research should include a comprehensive evaluation of both clinical and radiographic outcomes to ensure a more complete comparison of surgical to conservative management strategies in paediatric femoral shaft fractures.

It is important to note that while TEN offers the benefit of earlier functional recovery, it is not without drawbacks. TEN requires general anaesthesia, intraoperative imaging, surgical expertise and access to hospital infrastructure, factors that may not be readily available in all healthcare settings, particularly in resource-constrained environments. Moreover, TEN usually necessitates a second surgery for implant removal, which may increase costs and expose the child to additional anaesthesia-related risks. In contrast, hip spica casting remains a non-operative, low-cost alternative that avoids surgical risks and is more feasible in many low-resource settings.

These differences in invasiveness, cost, anaesthetic requirements and parental preference were not the focus of the current study but are critical factors in treatment planning and should be carefully weighed when choosing the most appropriate management strategy. Future studies should incorporate these dimensions to offer a more holistic assessment of both treatment modalities.

While our study was strengthened by prospective data collection and standardised follow-ups, limitations include a relatively small sample size, short duration of follow-up and single-centre design. Additionally, a few patients were lost to follow-up, reducing the overall cohort. Larger, multicentre randomised controlled trials are needed to validate these results and provide more robust evidence to guide treatment decisions.

## Conclusions

This study observed that titanium elastic nailing was associated with the earlier initiation of weight bearing compared to hip spica casting in children aged 6-12 years with femoral shaft fractures. As hip spica casting is more commonly recommended for younger children under the age of five, our findings suggest that titanium elastic nailing may represent a more appropriate option in older paediatric patients, particularly in settings where surgical infrastructure is available. While TEN demonstrated potential advantages in facilitating earlier functional recovery, these results should be interpreted within the specific context of the study's focus on time to weight bearing and hospital stay.

## Appendices

The severity of femoral shaft fractures was categorised using the Winquist classification, based on radiographs of the anteroposterior and lateral views of the thigh [19]. This is presented in Table 4.

**Table 4:** Winquist classification of femoral shaft fractures as described in radiographic assessments

Winquist Type	Description	Radiographic Assessment
Type 0	No comminution or a small butterfly fragment less than 25% of the width of the bone	Assessed on anteroposterior and lateral radiographs of the thigh
Type I	Small butterfly fragment less than 25% of the width of the bone	Assessed on anteroposterior and lateral radiographs of the thigh
Type II	Butterfly fragment 50% or less of the width of the bone	Assessed on anteroposterior and lateral radiographs of the thigh
Type III	Comminuted with a large butterfly fragment, greater than 50% of the width of the bone	Assessed on anteroposterior and lateral radiographs of the thigh
Type IV	Severe comminution of an entire segment of the bone (segmental comminution)	Assessed on anteroposterior and lateral radiographs of the thigh
